# Application of Fluorescein Fluorescence in Vascular Neurosurgery

**DOI:** 10.3389/fsurg.2019.00052

**Published:** 2019-09-18

**Authors:** Xiaochun Zhao, Evgenii Belykh, Claudio Cavallo, Daniel Valli, Sirin Gandhi, Mark C. Preul, Peter Vajkoczy, Michael T. Lawton, Peter Nakaji

**Affiliations:** ^1^Department of Neurosurgery, Barrow Neurological Institute, St. Joseph's Hospital and Medical Center, Phoenix, AZ, United States; ^2^Department of Neurosurgery, Irkutsk State Medical University, Irkutsk, Russia; ^3^Department of Neurosurgery, Charité – Universitätsmedizin Berlin, Berlin, Germany

**Keywords:** fluorescein angiography, fluorescein fluorescence, fluorescein sodium, vascular neurosurgery, aneurysm, arteriovenous malformation, arteriovenous fistula

## Abstract

**Background:** Fluorescein sodium (FNa) is a fluorescent drug with a long history of use for assessing retinal blood flow in ophthalmology; however, its application in vascular neurosurgery is only now gaining popularity. This review summarizes the current knowledge about using FNa videoangiography in vascular neurosurgery.

**Methods:** We performed a literature review on the usage of FNa for fluorescent videoangiography procedures in neurosurgery. We analyzed methods of injection, dosages of FNa, visualizing platforms, and interpretation of FNa videoangiography. We also reviewed practical applications of FNa videoangiography during various vascular neurosurgeries.

**Results:** FNa videoangiography can be performed with intraarterial (intracarotid) or intravenous dye injections. Both methods provide excellent resolution with enhanced fluorescence that shows intravascular blood flow on top of visible surrounding anatomy, and both allow simultaneous purposeful microsurgical manipulations. Although it is invasive, an intracarotid FNa injection results in faster contrast appearance and higher-intensity fluorescence and requires a lower dose per injection (reported range, 1–50 mg) compared with peripheral intravenous FNa injection (reported range, 75–2,000 mg or 1–1.5 mg/kg body weight). Four optical excitation/detection tools for FNa videoangiography have been successfully used: conventional xenon-light operating microscope with a special filter set, pencil-type light-emitting diode probe with a filter set, laser-illumination operating microscope, and an endoscope with a filter set. FNa videoangiography was reported to be feasible and useful in various clinical scenarios, such as examining the feeders and drainers in arteriovenous malformation surgery, checking the patency of a microvascular anastomosis, and assessing blood flow during aneurysm clipping. FNa videoangiography can be repeated during the same procedure and used along with indocyanine green (ICG) videoangiography.

**Conclusions:** Compared with ICG videoangiography, FNa videoangiography has the advantages of enabling real-time inspection and better visualization at deep locations; however, thick vessel walls limit visualization of FNa in larger vessels. FNa videoangiography is a useful tool in multiple neurovascular scenarios and merits further studies to establish its clinical value.

## Introduction

Techniques for evaluating blood flow are essential for successful neurosurgery on vascular lesions. Vascular lesions, such as cerebral aneurysms, arteriovenous malformations (AVMs), and dural arteriovenous fistulas (DAVFs), and other clinical scenarios requiring vascular anastomosis are characterized by unique flow alterations (e.g., aneurysmal turbulent flow). Clinical outcomes may be catastrophic if blood flow is severely compromised or if a vessel is ruptured during these procedures. Surgical intervention in such lesions includes blood flow monitoring to minimize the risk of rupture or to reestablish compromised normal circulation.

Multiple methods have been developed to examine circulation intraoperatively. These methods can be broadly classified on the basis of the principles of physics, drugs, and the devices used.

1. Observation and palpation, using the classic milking test, can assess patency of a dissected vessel. Pulsation waves and the color of blood (bright-red oxygenated vs. dark-red deoxygenated) are qualitative signs that are helpful in flow assessment but have multiple limitations (1).
2. Contact probe-based Doppler angiography can measure the volume and speed of the blood flow (2) using either a point-probe, which measures the target artery from one side, or an intracranial Charbel Micro-Flow probe (Transonic, Ithaca, NY), which measures the target artery from two sides.
3. Wide-field laser speckle imaging (785 nm, 50 mW laser) allows the generation of a real-time two-dimensional color-coded map of perfusion during open surgery and can be used as a noninvasive visualization and measurement of the relative cortical blood flow (3).
4. Digital subtraction angiography (DSA) is a robust but invasive imaging technique that remains the gold-standard examination for many vascular lesions. Intraoperative DSA requires a hybrid room setting (angiography setup in the operating room) and separate sterilization area (i.e., femoral area) (4–6).
5. Fluorescent videoangiography with various wide-field surgical microscopes with appropriate filters includes the use of indocyanine green (ICG) videoangiography and fluorescein sodium (FNa) videoangiography.
6. Confocal laser endomicroscopic angiography can employ FNa or ICG and assess brain microvasculature.

This paper is a focused review of the history and current applications of FNa videoangiography for intraoperative neurovascular imaging.

## History of FNa Videoangiography

According to Van Cader (7), the earliest study with FNa was conducted in 1881 by Ehrlich and colleagues, who analyzed the distribution of FNa after intravenous (IV) injection and demonstrated its presence in the anterior chamber of the eye. In ophthalmology, FNa has been extensively used to assess retinal blood flow (8, 9). In the late 1940s, FNa was adopted by Moore et al. to visualize neoplastic tissue in gastric adenocarcinomas and brain tumors (10, 11). Glial tumors were found to produce the most consistent positive fluorescence.

In 1967, FNa was first used to evaluate the intracranial circulation in animals and humans by Feindel et al. (12). A report of an AVM surgery by the same group in 1971 was the first record of FNa application in vascular neurosurgery (13). Since then, the number of studies reporting FNa videoangiography has been relatively limited, with the majority of studies published after 2013 (6, 14–23).

## Metabolism and Safety of FNa

Upon IV administration, FNa loosely binds to plasma proteins and exists as a protein-bound (80%) and free-salt (20%) substance. FNa is metabolized by glucuronidation in the liver. About 80% of IV FNa is converted to a monoglucuronide within 1 h, which is 95% less fluorescent. FNa is rapidly cleared by the kidneys through filtration and secretion and is almost completely excreted by 24 h (24).

FNa has been shown to be minimally toxic at a dose of 20 mg/kg body weight (1,500 mg for a 75-kg patient) (25). In ophthalmology, however, FNa is used extensively at dosages of 500 mg (26, 27) and as much as 30 mg/kg body weight. Median lethal doses of IV FNa are 2,200 mg/kg for mice, 600 to 1,000 mg/kg in rats, 1,000 mg/kg in dogs, and 350 mg/kg in rabbits (28). Complications such as cardiac effect, respiratory reaction, or seizure have been reported rarely, with the frequency of severe adverse effects reported as 1 in 1,900 and death as 1 in 222,000 (29). Patients injected with doses of more than 5 mg/kg body weight exhibit temporary yellow staining of skin, which disappears within 6 to 24 h as FNa is cleared (10). It was noted that patients with severe liver diseases may experience prolonged yellow skin staining (10). FNa administration for vascular neurosurgical applications requires a relatively low dose, and no complications related to FNa have been reported in the neurosurgical literature.

## Dosages of FNa for Vascular Applications

IV and intraarterial (IA) injections of FNa require different dosages for optimal vascular imaging. IV administration usually requires a higher dosage than IA injection to achieve good contrast, as FNa dilutes during peripheral circulation, and 80% of the FNa binds to albumin and is deactivated in this process (30, 31). Wrobel et al. (32) reported a case of high-dose FNa videoangiography using 2,000 mg IV bolus. In other neurosurgical reports, IV doses range from 75 mg to 500 mg per bolus (6, 18–21, 30, 31, 33) or from 1 mg/kg to 1.5 mg/kg per body weight (16, 34).

Doses for IA injection are low, with 2 studies from the 1970s reporting 10 mg (13) and 50 mg (33) IV boluses, and more recent studies have used a dose as low as 10 ml of 0.01–0.02% FNa solution (0.001–0.002 mg) (20, 31) and 5 ml of 0.5–1% FNa solution (0.025–0.05 mg) (17). After investigating different dosages of FNa, Kuroda et al. reported 10 ml of 0.01–0.02% FNa bolus (0.001–0.002 mg) can be used to sufficiently detect the parent artery and perforating arteries of aneurysms without staining the vessel wall and without persistence in the vessel 5 min after IA injection (31).

## Site of Injection: IV or IA

IV routes can use central or peripheral venous access, which usually has not been specified in reports. IA injection, however, needs to be made directly into the carotid or vertebral circulation. Thus, the peripheral arterial accesses (radial artery) for blood pressure monitoring would be no different with an IV injection in terms of FNa mixing with blood. However, IA injection requires an additional vascular access site with associated risks, which is a drawback.

Catheterization of the common carotid artery (CCA) may be performed via a direct transcutaneous puncture or transfemoral retrograde catheterization. However, the detailed technique used to access the CCA for FNa injection was not specified in majority of the literature (13, 20, 31, 33). Ichikawa et al. inserted an IA catheter from the superficial temporal artery into the CCA for approximately 5 to 10 cm in a retrograde fashion and then successfully performed FNa videoangiography (17). Other potential retrograde injection sites, such as the occipital artery, merit future feasibility studies.

**Figure 1:**
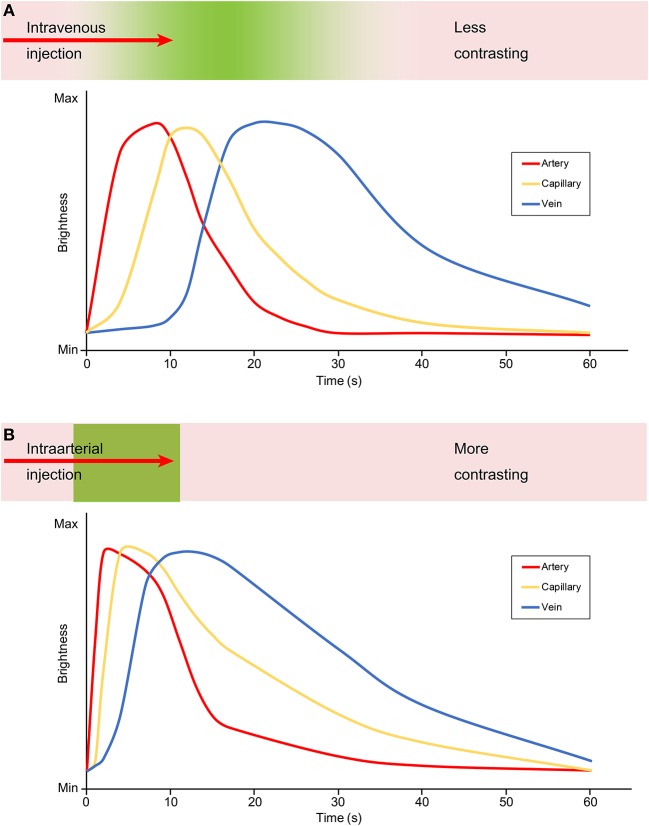
Graphic demonstrating the contrast and brightness changes during angiography with an **(A)** intravenous and **(B)** intraarterial FNa injection. The green color embedded in the bar above the graph represents the flow of the fluorescein. With an intravenous injection, the fluorescein is diluted and contrasts less as shown in the less steep and delayed graph, compared with an intraarterial injection during which the fluorescein is more concentrated and contrasts better as shown by the steep and acute graph. Max, maximum; Min, minimum. Used with permission from Barrow Neurological Institute, Phoenix, Arizona.

The major difference between IV injection and IA injection of FNa is the time period from the injection to different phases of FNa appearance in the field of view (Figure 1). The arterial phase of IA FNa injection appears immediately after injection (20, 31), usually no more than 2 s (13), and the venous phase fades out in <1 min (17). In comparison, the appearance takes longer in both phases with IV injection—it usually takes at least 10 s for the arterial phase and a minimum of 5 min until the fluorescence fades out (17, 31). The show-up and the fade-out of each phase after an IV injection are steep, and those in IV injection are delayed.

A shorter time period until fade-out is essential if repeated angiography is required, such as for evaluation of a DAVF before and after ligation or when accessing the patency of perforators during a series of clip adjustments in an aneurysm surgery (31). Kuroda et al. reported that IA FNa videoangiography can be repeated at least 5 times without staining the vessel wall in a short period of time, with the total dose significantly below the safety limits (31). However, the length of the period of intravascular stay was under debate; Kakucs et al. advocated that the longer time window of intravascular stay is one of the advantages of the IV injection, which provides more time of the angiography for use in analysis (15).

Because 80% of FNa molecules combine with albumin (30, 31), IV FNa injections usually require a higher dose than IA injections, which may lead to vessel wall staining and decrease the intravascular-extravascular contrast (31, 33). Furthermore, even with a higher IV dose administered, the observed fluorescence decreases as FNa dilutes with blood along the circulation. In IA injection, however, the FNa concentration in cerebral vasculature is high, providing higher contrast to the background anatomy. This is established for both FNa videoangiography (Figure 1) and ICG videoangiography (20, 31, 35). However, both IA and IV FNa administration routes can provide sufficient fluorescence intensity and sufficient information for interpretation (20).

In summary, compared with IV injection of FNa, IA injection has the advantage of better contrast, lower required dose, and the ability to be repeatedly performed in a limited time period without staining to the vessel wall. The disadvantage of IA injection is the necessity of additional vascular access to the CCA and its associated risks. Longer intravascular stay may be interpreted differently and can be advantageous or disadvantageous for both methods of administering FNa.

## Phases of FNa Videoangiography and Factors That Affect Phases

As with any angiography, FNa videoangiography has three main phases in which certain vessels are characteristically highlighted by fluorescence: the arterial, capillary, and venous phases. The duration of these phases (i.e., the duration of FNa fluorescence in arterial, capillary, and venous beds) depends on multiple factors. Mindful consideration of these factors is crucial for assessment and differentiation of normal and pathologic conditions. The interrelation of various factors, such as dosage and the route of administration, adds another level of complexity to assessments. Below, we briefly list these factors and discuss those particularly relevant to FNa in more details.

### Site of Injection

With IV injections, FNa travels in the cardiopulmonary circulation and then appears in the systemic arterial circulation, which allows FNa to mix with large quantities of blood and thus provides a longer time to peak and less bright fluorescence compared with an IA injection. Additionally, the speed of injection plays a role in the fluorescence phases and should be standardized as a rapid bolus injection.

### Dose

A large dose can prolong the intravascular stay of FNa but has the disadvantage of unspecific vessel wall staining. A dose of 2,000 mg of FNa was reported to remain detectable in the cerebral vessels for 60 min, and the authors suggested that using a lower dose may be better, as used with repetitive angiographies (32).

### Vessel Wall Staining

Detected intravascular fluorescence can be due to FNa in the blood or due to the staining of the vessel wall. Stained vessel walls can lower the contrast effect and affect the interpretation of the videoangiography, especially given the impact on quality of subsequent angiographies.

### Vascular Abnormalities

The arterial phase appears early when used in a patient with an abnormal connection between the venous and arterial circulations, such as a DAVF or AVM, where the blood flow is aberrantly fast. The arterial phase was reported to be 11 s in a case report of a DAVF (34) and 10–15 s in AVM surgeries (13, 16, 18, 23) after IV FNa injection. These durations are shorter than those reported during IV FNa videoangiography in aneurysm surgeries, in which the arterial phase typically appears for more than 20 s (20, 22, 31, 32).

The effect of a bypass can also influence the arterial phase appearance. Little et al. reported an average 1.7-s time decrease from the injection to the arterial phase in cortical arteries after superficial temporal artery–middle cerebral artery bypass. This change, along with visual transit of fluorescence signal through an anastomosis, can be used as an indicator of a successful bypass (33).

### Cardiovascular Parameters

Cardiovascular parameters, such as arterial blood pressure and heart rate, may affect time-dependent angiographic parameters and should be taken into consideration.

### Visualization Platform Parameters

The focus distance of the microscope, its angulation, position within the field of view, photobleaching properties, and irregularities of the surgical field increase the complexity when assessing fluorescence images (36).

## Visualizing Platforms

**Figure 2:**
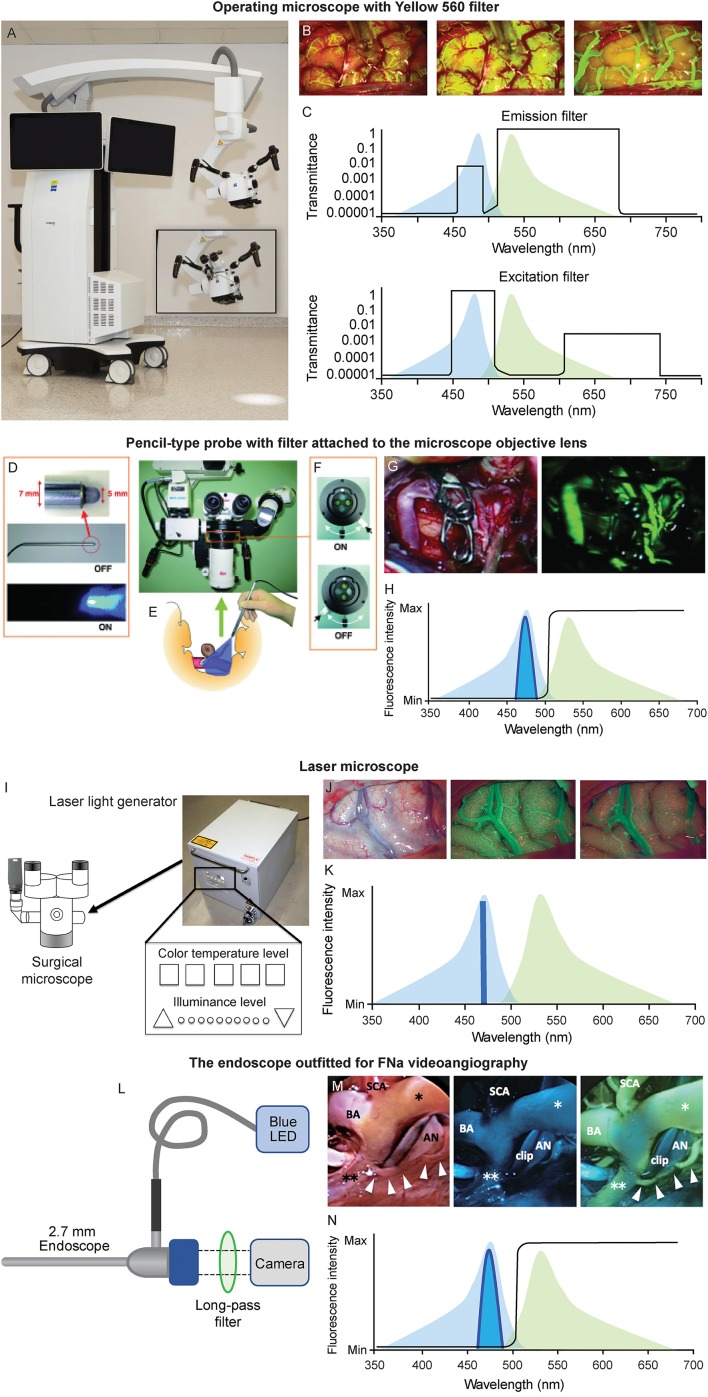
Various platforms used for FNa videoangiography. **(A–C)** Operating microscope. **(A)** The operating microscope with Yellow 560 module (ZEISS KINEVO 900, Carl Zeiss AG, Oberkochen, Germany). **(B)** Intraoperative views of FNa videoangiography (left to right: arterial, capillary, and venous phases). **(C)** Patented filter combinations used in the operative microscope balances the intensities of narrow bands of excitation and emitted light to create a clear operating field view with yellow fluorescence of fluorescein. The excitation (bottom) and emission (top) profiles of fluorescein (peaks are 485 nm, blue; 514 nm, green, respectively) are shown on the background. The transmittance of filters at various wavelengths results in the uniform intensity of all bands, with a higher intensity of emitted yellow light. **(D-H)** Pencil-type probe with filter attached to the microscope objective lens. **(D)** The pencil-type probe with blue LED emission. **(E)** Sketch demonstrating the intraoperative usage of the probe. **(F)** The switch of the filter adapted to the operating microscope. **(G)** The intraoperative view using this device (left, fluorescence off; right, fluorescence on). **(H)** A diagram showing optical setup of the device. Blue LED light is concentrated around 465 nm, and a long-pass filter (black line) allows green emission light into the camera. **(I-K)** The laser microscope. **(I)** The illuminating device. **(J)** Images of the laser FNa videoangiography (left to right: intraoperative image under white light, capillary phase and venous phases of the FNa videoangiography). **(K)** Regular excitation (light blue) overlapping the emission (light green) laser light contains light only at 464 nm wavelength and does not interfere with the emission light; thus, the videoangiogram has good contrast. **(L–N)** An endoscope outfitted for FNa videoangiography. **(L)** A 2.7-mm straight endoscope is connected to a blue LED light source and a long-pass filter is inserted at the camera attachment. **(M)** The intraoperative view of endoscopic FNa videoangiography (left to right: under white light, under the blue LED before FNa injection, and after FNa injection). **(N)** The diagram showing optical setup of the device. Blue LED light is concentrated around 465 nm, and a long-pass filter (black line) allows green emission light into the camera. *, right posterior cerebral artery; **, left posterior cerebral artery; AN, aneurysm; BA, basilar artery; Max, maximum; Min, minimum; SCA, superior cerebellar artery. **(A–C,H,K,L,N)** are used with permission from Barrow Neurological Institute, Phoenix, Arizona. **(D–G)** Are used with permission from Suzuki et al. (37). **(I)** is copyright of Sato et al. (38) and made available under Creative Commons. **(J)** is used with permission from Ito et al. (22). **(M)** is used with permission from Hashimoto et al. (20).

Different visualizing platforms that have been developed for FNa videoangiography are described briefly below (Figure 2).

### Conventional Operating Microscopes With Fluorescein Filters (Figures 2A–C)

Unlike ICG videoangiography, which requires a separate infrared camera attached to the operating microscope to detect the infrared emission signal (39), the emission light of FNa requires only a filter for fluorescence to be visualized within the visible light spectrum. This may be the reason for the delayed application of ICG into the vascular field (39).

An FNa-specific filter set can be integrated into the surgical microscope to guarantee optimal intraoperative visualization of fluorescence and images superior in clarity. Novel microscopes have improved so much in fluorescence modality, and FNa fluorescence can offer such a brighter view that tumor resection can be performed in fluorescence mode throughout the whole surgery rather than switching between visualization modalities (40).

### Pencil-Type Probe With Detachable Filter for the Microscope (Figures 2D–H)

Suzuki et al. used a pencil-type probe with a blue light-emitting diode (LED) at the tip (20, 30, 31); this setup can offer excitatory light while FNa fluorescence can be observed via a filter attached to the microscope (20, 30, 31). This pencil-type probe and filter represent an affordable option, and it enables surgeons to equip existing surgical microscopes with an FNa visualization mode.

A custom long-pass filter inset was designed to be manually installed at the bottom of the surgical microscope cylinder to visualize the FNa fluorescence (41). This device includes optical filters positioned across the three light pathways: two barrier filters for observation within the wavelength of FNa emitted fluorescence and one excitation filter.

### Laser Illumination-Based Operating Microscope (Figures 2I–K)

Ito et al. reported the application of the laser microscope for intraoperative fluorescence cerebral angiography (22). Unlike traditional operating microscopes with a xenon lamp-generated light (wide wavelength spectrum), the laser microscope contains only three lasers of 640 nm (red), 532 nm (green), and 464 nm (blue) wavelengths. During illumination, these three wavelengths crosswire, resulting in white light that is similar to the regular xenon-generated light. During FNa videoangiography, the system turns off the green laser while leaving on the blue laser and low-intensity red laser. The peak emission wavelength of the FNa is 520 nm, which does not overlay with reflected blue and red laser lights. Therefore, such illumination offers better contrast. Furthermore, the laser illumination-based microscope produces significantly less tissue heat compared to xenon lamp illumination (38). Although this laser microscope is an early prototype, it provides well-controlled illumination that may benefit future developments of surgical microscopes.

### FNa Videoangiography With an Endoscope (Figures 2L–N)

Hashimoto et al. reported their experience of FNa videoangiography visualized through the endoscope (20). A blue LED was adapted to the light source of the 2.7-mm endoscope to offer excitation light, and the emission light passed through a long-pass filter and was received by a camera.

In their detailed report, only three of 18 aneurysms could be completely inspected under the microscope after clipping, and no neck remnant was confirmed. Although microscopic FNa videoangiography showed that in all cases complete aneurysm occlusion had been achieved, subsequent inspection with the endoscopic FNa videoangiography allowed the investigators to detect and correct three of 18 cases with neck remnants and two of 18 cases with incomplete occlusion. Moreover, the perforator origin could be visualized only via endoscopic inspection and not with the microscope, which allowed the identification and correction of two perforator occlusion events. The authors concluded that endoscopic FNa videoangiography had the same accuracy as DSA.

Although ICG videoangiography can also be obtained via endoscope, such ICG-infrared compatible endoscopes exceed 4 mm in diameter (42–44). Hashimoto et al. reported that in nine of 29 cases, a 4-mm endoscope was too large for visualization at deep locations (20). Therefore, thin endoscopes capable of FNa videoangiography represent a valuable adjunct to visualize blood flow in the vessels that are obscured during wide-field operations using microscope-based angiography.

## Comparison of ICG and FNa Videoangiographies

In vascular neurosurgery, intraoperative DSA remains the gold standard to assess the blood flow, but DSA requires a hybrid operating room and increases operating time (19).

Although ICG was applied to assess cerebral blood flow much later than FNa [ICG in 2003 (39), FNa in 1967 (12)], cerebral fluorescent videoangiography is currently more common with ICG than with FNa. ICG videoangiography can be repeated quickly and can demonstrate the vasculature clearly (45, 46); however, ICG still has several drawbacks. FNa and ICG both have features that can provide benefits in different scenarios—the features of both fluorophores are summarized in Table 1.

**Table 1:** Comparison of Advantages and Disadvantages of ICG- and FNa-Based Videoangiography.

**Parameter**	**Fluorescent angiography**	
	**ICG**	**FNa**
Thick vessel wall	Deeper penetration depth; can visualize STA, ICA, VA	Shallow penetration depth; cannot show flow in ICA or VA
Small vessels	Harder to visualize	Easier to visualize
Repeated IV injections or increased dose	Decreased intensity because of quenching	Can stain vessel wall resulting in false-positive fluorescence
Fluorescence detection	Infrared camera only	Eye, camera
Display	Always digital signal; separate LCD display or image injection into microscope eyepieces	Viewed through eyepieces or on any display
On-the-fly angiography	Requires image overlay; otherwise, need to review ICG video separately from natural light view	Can be in real time
View of surgical field	Two-dimensional	Three-dimensional in eyepieces/display
Contrast	Very high contrast	Average to high contrast

*FNa, fluorescein sodium; ICA, internal carotid artery; ICG, indocyanine green; STA, superficial temporal artery; VA, vertebral artery*.

On one hand, unlike FNa, which can be observed in oculars, ICG requires a dedicated camera to detect the near-infrared signal and a separate display to present a pseudo-colored signal. The ICG signal does not interfere with visible spectral reflectance from the operating field and therefore provides a better contrast than FNa between surrounding structures (signal always = 0) and intravascular ICG (signal is high and always > 0), without the need to sacrifice any wavelength of the visible spectrum.

On the other hand, FNa can be seen as an orange color by the naked eye or as a bright yellow color when fluorescent. Because the excitation and emission wavelengths of FNa are within the visible spectrum, creation of a high contrast for FNa visualization requires the user to sacrifice some portion of the visible spectrum. Improvements with optical filter technologies have made it possible to selectively attenuate or block multiple spectral bands, allowing brighter overall images and making the consequential image demonstrate most of the reflected visible colors (brain, blood, and other tissues) and brighter FNa fluorescence simultaneously.

ICG is a fluorophore that can be visualized only by a near-infrared detector with a complete dark background (45), in which case the relationship of the surrounding anatomy and the vessels cannot be demonstrated. Furthermore, the ICG imaging can be visualized only via a separate infrared camera; therefore, the surgeon cannot change the view or perform real-time dissection on the basis of the information obtained from ICG videoangiography. Although these limitations were partially solved by live ICG-image overlay features on operating microscopes (47), such microscopes are not yet widely available.

FNa videoangiography provides less contrast than ICG but has the advantage of allowing visualization of the angiogram embedded in the thee-dimensional background anatomy within the ocular lens, which can enable real-time manipulation in situations of inadequate exposure, clip adjustment, and so on.

The second ICG injection can be less clear and less contrasting than the first because of quenching effects of dye remaining from the first injection (31). However, higher dosages of FNa with IV injection may stain the vessels for a substantial time, decreasing the specificity of FNa signal. Therefore, in terms of repeating fluorescent videoangiography quickly, ICG videoangiography may be preferred over IV FNa videoangiography (19, 21).

Another advantage of ICG is the longer wavelength emission that could penetrate thick vessel walls. It is reported that ICG is favorable in visualizing major arteries, such as the internal carotid artery and superficial temporal artery (21).

Lane et al. performed a prospective study comparing ICG and FNa videoangiographies in 22 patients and drew the conclusion that FNa can provide better visualization of vasculature at high magnification within deep operative fields (19). The limitation of ICG visualization at deep operative fields has been mentioned as its drawback in multiple studies (48–51). One study reported that inadvertent occlusion of small perforators was encountered in 6% (15/239) of the aneurysm clipping cases despite using ICG videoangiography (49).

ICG videoangiography and FNa videoangiography are valuable and mutually complementary tools in vascular neurosurgery, each with subtle advantages and disadvantages, and neither can yet replace intraoperative DSA as the gold standard. FNa and ICG could be used together sequentially as they do not interfere with each other. As technologies advance, the disadvantages of either modality are becoming less substantial (e.g., real-time ocular overlay of ICG videoangiography and laser illumination system).

## Clinical Application of FNa Videoangiography in Vascular Neurosurgery

The use of FNa videoangiography has been reported in many types of vascular neurosurgery, including treating aneurysms, AVMs, and DAVFs and bypass surgeries. A summary of clinical studies is presented in Table 2 (6, 13, 15–23, 30–34), and clinical applications of FNa videoangiography are discussed below.

**Table 2:** Summary of clinical application of fluorescein videoangiography in the literature.

**References**	**Injection method**	**Visualizing platform**	**Dose/concentration**	**Cases number**	**Lesion type**	**Time to arterial phase**	**Time to fade out**	**Comments**
Feinde et al. (13)	IA	Stroboscopic light with Wratten 2B filter	1–2 ml/1% (10–20 mg)	1	AVM	Less than 2 s	N/A	First report in the literature
Little et al. (33)	IA (CCA)	Strobe light with Kodak-Wratten 47A filter	5 ml/1% (50 mg)	15	STA-MCA bypass	2.4 ± 0.4 s pre-bypass; 0.7 ± 0.3 s post-bypass	N/A	Bypass led to a 1.7 s decrease of the period from injection to arterial phase.
Wrobel et al. (32)	IV	ILC 302 and Oriel liquid light with Wratten 47A filter	20 ml/10% (2 g)	1	Aneurysm	30 s	60 min	2 g FNa caused long-time vessel wall staining
Suzuki et al. (30)	IV	A pencil-type probe with a blue LED/ filter attached to microscope	5 ml/10% (500 mg)	23	Aneurysm	15 s	N/A	The pencil-type probe is an affordable option for FNa videoangiography
Kuroda et al. (31)	IV and IA	A pencil-type probe with a blue LED filter attached to microscope	IV 5 ml/10% (500 mg); IA 10 ml/0.01–0.02% (1–2 mg)	IV, 5; IA, 13	Aneurysm	IV, 30 s; IA, immediately	IV, minimally 5 min; IA, 30 s	First study to report the advantage and disadvantage of IV and IA injections
Rey-Dios and Cohen-Gadol (16)	IV	OPMI Pentero 900	1 mg/kg	2	Aneurysm, 1; AVM, 1	20 s	N/A	
Ichikawa et al. (17)	IA (STA)	OPMI Pentero 900	5 ml/0.5–1%	10	Aneurysm	Immediately	Less than 1 min	3 F catheter inserted 5–10 cm into the STA
Lane and Cohen-Gadol (18)	IV	OPMI Pentero 900	75 mg	4	AVM	10–15 s	N/A	FNa videoangiography has the “real-time inspection” feature which is valuable in AVM surgery
Lane et al. (19)	IV	OPMI Pentero 900	75 mg	22	Aneurysm	20 s	20–30 min	Prospective comparison of FNa and ICG
Misra et al. (34)	IV	N/A	1.5 mg/kg	1	DAVF	11 s	N/A	First case report of use with a spinal AVM
Hashimoto et al. (20)	IV and IA	Olympus endoscope with long-pass filter; blue LED at the light source	IV 5 ml/10% (500 mg); IA 10 ml/0.01–0.02% (1–2 mg)	IV, 5; IA, 13	Aneurysm	IV, 30 s; IA, immediately	IV, N/A; IA 30 s	Endoscope can offer more information such as neck remnant and perforators preservation
Kakucs et al. (15)	IV	OPMI Pentero 900	5 ml/10% (500 mg)	10	Aneurysm	15 s	N/A	
Matano et al. (21)	IV	OPMI Pentero 900	250 mg	23	Aneurysm 18; bypass, 5	NA	N/A	Comparison of ICG and FNa in vascular surgery
Ito et al. (22)	IV	M500 OHS1 Laser microscope	2.5 ml/10% (250 mg)	1	Aneurysm	20 s	N/A	A novel laser microscope examining fluorescein angiography
Narducci et al. (6)	IV	OPMI Pentero 900	500 mg	11	Bypass	15–20 s	20–25 min	
Serrano-Rubio et al. (23)	IV	OPMI Pentero 900	75 mg	12	AVM	11.6 s	N/A	

*AVM, arteriovenous malformation; CCA, common carotid artery; DAVF, dural arteriovenous fistula; FNa, fluorescein sodium; IA, intraarterial; ICG, indocyanine green; IV, intravenous; LED, light-emitting diode; MCA, middle cerebral artery; N/A, not applicable; STA, superficial temporal artery*.

### Cerebral Aneurysm Surgery (Figure 3)

For cerebral aneurysm surgery, it is of paramount importance to confirm the obliteration of the aneurysm neck as well as the patency of the parent vessels and small perforators intraoperatively (31). Lane et al. reported that FNa is preferable over ICG in terms of visualizing small perforators and aneurysm obliteration (19). For aneurysms at deep locations requiring a narrow corridor (i.e., anterior cerebral artery aneurysms through the lateral frontal approach), real-time manipulation of the surrounding anatomy under FNa videoangiography allows maximum exposure and inspection of the vessels of interest (19).

**Figure 3:**
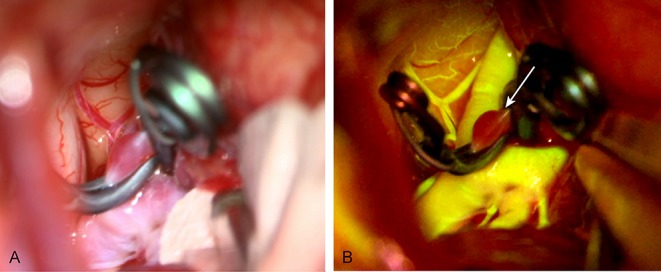
Intraoperative images of an unruptured anterior communicating artery. **(A)** Intraoperative image under normal light. **(B)** Intraoperative image taken during FNa videoangiography; the aneurysm is not filling after being clipped (white arrow). Used with permission from Barrow Neurological Institute, Phoenix, Arizona.

As discussed above, the endoscope can offer more information than FNa angiography, such as location of neck remnants and perforator preservation, as the endoscope provides a close and multi-angled inspection (20).

### AVM Surgery (Figure 4)

AVMs are usually adhesive to the surrounding brain parenchyma. AVMs can be regarded as “intra-axial lesions” as most require subarachnoid and pial dissection. Numerous feeders can supply the nidus from the deep side, and these feeders may be blocked by brain parenchyma. Such a complex nature means that DSA remains the gold standard for evaluation (52), but it is not always available.

**Figure 4:**
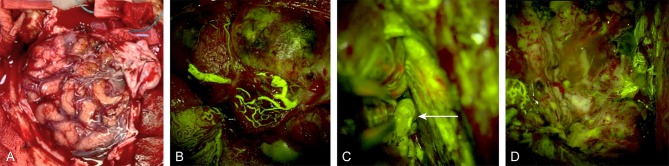
Intraoperative images of a left frontal arteriovenous malformation **(A)** under normal light, **(B)** during FNa videoangiography, **(C)** during real-time inspection of the nidus and the deep feeders (white arrow) under FNa videoangiography during dissection, and **(D)** after total removal of the malformation. Used with permission from Barrow Neurological Institute, Phoenix, Arizona.

As mentioned above, fluorescence with traditional ICG videoangiography cannot be visualized via an ocular in real time. Deep feeders and the adhesive nature of AVMs make them challenging to operate on because thorough information usually cannot be obtained from a single angiography. Especially after circumferential dissection of the nidus or ligation of the superficial feeders, a premature venous filling indicates the presence of remaining deep feeders. Real-time dissection of the nidus and full inspection around it under videoangiography can facilitate localizing the rest of the feeders as well as provide a thorough examination of the lesion (16, 18). The real-time feature of FNa videoangiography is preferable over ICG in such scenarios. In the only case of a spinal AVM reported as treated under FNa videoangiography (34), the vascular nidus was well visualized as being embedded in the background anatomy and the AVM was successfully resected.

### DAVF Surgery

To date, there is no report of the application of FNa videoangiography for treating a DAVF. Unlike AVMs, DAVFs do not adhere to the surrounding parenchyma. The ligation of the fistula is concise and quick if the fistula can be accurately located; this feature of treating a DAVF requires repeated angiography in a short period of time. Even though IV injection of FNa for videoangiography has a relatively long intravascular stay, IA injection might be suitable in this scenario, and future investigation is merited.

### Cerebrovascular Bypass Surgery (Figure 5)

FNa videoangiography can be used to evaluate the patency of the anastomosis and the improvement of the stenosis during cerebrovascular bypass surgery. The feature of real-time manipulation does not make FNa videoangiography differ from the ICG videoangiography in the bypass assessment (6). However, IA injection can be used to evaluate the effectiveness of the bypass apart from the evaluation of the patency. Little et al. carried out a study using IA injection in 1979 and reported that the duration from the injection to the arterial phase decreased from 2.4 ± 0.4 s to 0.7 ± 0.3 s (33). Such a decrease in time can help evaluate the improvement of the ischemic condition and offer another supplementary tool for evaluating the success of the bypass surgery.

**Figure 5:**
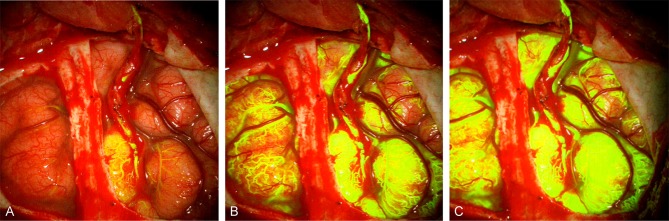
FNa videoangiography images from a patient with moyamoya disease after a superficial temporal artery to middle cerebral artery bypass shows filling of **(A)** donor and recipient arteries, **(B)** cortical capillaries, and **(C)** all cortical vascular networks. Notice the filling starts at the bypass site and spreads around. Used with permission from Charite Universitätsmedizin Berlin, Berlin, Germany.

## Conclusion

The FNa-based fluorescent videoangiography techniques reviewed here represent an armamentarium of useful tools for various types of vascular neurosurgeries. Compared with ICG videoangiography, FNa videoangiography has the advantage of three-dimensional visualization of surrounding anatomy and allows real-time surgical manipulation, especially of small vessels in a narrow field. The disadvantages of FNa videoangiography are the incompetence in visualizing flow in thick-walled vessels and the staining of vessel walls at high doses. Advanced microscopy technologies, such as endoscopy and laser microscopy, have the potential to further improve the utility of FNa videoangiography. Finally, FNa videoangiography is affordable and may be complementary to ICG videoangiography for vascular neurosurgery.

## Author Contributions

PN and MP: conception and design. PN, ML, and PV: providing clinical cases. XZ: drafting the article. EB and CC: figures and graphs producing. EB, CC, DV, and SG: revising the article. PN: study supervision.

### Conflict of Interest Statement

The authors declare that the research was conducted in the absence of any commercial or financial relationships that could be construed as a potential conflict of interest.

## Abbreviations

AVM: arteriovenous malformation
CCA: common carotid artery
DAVF: dural arteriovenous fistula
DSA: digital subtraction angiography
FNa: fluorescein sodium
IA: intraarterial
ICG: indocyanine green
IV: intravenous
LED: light emitting diode.
